# Dietary Pattern Is Associated with Obesity in Older People in China: Data from China Health and Nutrition Survey (CHNS)

**DOI:** 10.3390/nu7095386

**Published:** 2015-09-23

**Authors:** Xiaoyue Xu, John Hall, Julie Byles, Zumin Shi

**Affiliations:** 1 Priority Research Centre for Gender, Health and Ageing, Hunter Medical Research Institute, School of Medicine and Public Health, the University of Newcastle, New Lambton Heights, NSW 2305, Australia; E-Mail: julie.byles@newcastle.edu.au; 2 Centre for Clinical Epidemiology and Biostatistics, Hunter Medical Research Institute, School of Medicine and Public Health, the University of Newcastle, New Lambton Heights, NSW 2305, Australia; E-Mail: john.hall@newcastle.edu.au; 3 School of Medicine, University of Adelaide, Adelaide, SA 5005, Australia; E-Mail: zumin.shi@adelaide.edu.au

**Keywords:** dietary pattern, obesity, central obesity, gender, urbanization levels, older Chinese people

## Abstract

Background: No studies have been conducted to explore the associations between dietary patterns and obesity among older Chinese people, by considering gender and urbanization level differences. Methods: We analyzed data from the 2009 China Health and Nutrition Survey (2745 individuals, aged ≥ 60 years). Dietary data were obtained using 24 h-recall over three consecutive days. Height, Body Weight, and Waist Circumference were measured. Exploratory factor analysis was used to identify dietary patterns. Multinomial and Poisson regression models were used to examine the association between dietary patterns and Body Mass Index (BMI) status/central obesity. Results: The prevalence of general and central obesity was 9.5% and 53.4%. Traditional dietary pattern (high intake of rice, pork and vegetables) was inversely associated with general/central obesity; modern dietary pattern (high intake of fruit, fast food, and processed meat) was positively associated with general/central obesity. The highest quartile of traditional dietary pattern had a lower risk of general/central obesity compared with the lowest quartile, while an inverse picture was found for the modern dietary pattern. These associations were consistent by gender and urbanization levels. Conclusions: Dietary patterns are associated with general/central obesity in older Chinese. This study reinforces the importance of a healthy diet in promoting healthy ageing in China.

## 1. Introduction

China is ageing rapidly. By the end of 2012, the population aged 60 years or above accounted for 14.3% of the total population. It is predicted that 25% of the population will be aged 60 years and over by 2035. The number of people aged 80 years and over is increasing even more rapidly than the 60–69 and 70–79 year age groups [1]. This change in the age structure in China will have significant impacts, including an increased prevalence of chronic non-communicable diseases (NCDs). NCDs have long duration with slow progression. The four main types of NCDs are cardiovascular diseases, cancers, chronic respiratory diseases, and diabetes [1,2]. Furthermore, recent studies point out that nonalcoholic fatty liver diseases (NFALD) precede the further development of metabolic syndrome and some NCDs, such as type 2 diabetes, especially in the older population [3,4].

China is also facing an obesity epidemic [5,6]. Obesity can be considered a chronic condition, as well as an important biological risk factor for NCDs [7]. During 1992 and 2011, among adults aged from 18 to 75 years, prevalence for overweight has dramatically increased from 14.6% to 45.4%, and has nearly tripled from approximately 5.2% to 15.1% for obesity [8]. In some areas, obesity rates have risen more than three-fold, which results from an increased consumption of more energy-dense, nutrient-poor food with high levels of sugar and saturated fats, combined with reduced physical activity [9].

Dietary patterns can be efficacious indicators of the impact of diet in health outcomes, as they illustrate the combined effects of diet intake. Studies have assessed dietary patterns and obesity among children and adolescents, but the results were not consistent [10,11]. Other studies have indicated dietary patterns are associated with the prevalence of cardiovascular risk factors, hypertension, obesity, body weight, and diabetes among Chinese adults [12,13]. However, research into the association between diet and NCDs among older Chinese is extremely scarce [14,15]. As older people have higher prevalence of NCDs, and have a higher risk of insufficient or unhealthy nutritional status [12,15], it is important to understand the effects of diet among older people. Thus, the aim of this study is to explore the association between dietary patterns and obesity among older Chinese. Moreover, our previous study showed that nutrition changes are linked to an increased burden of obesity and NCDs with considerable geographical variation [14]. Additionally, economic, demographic and related forces operating at different urbanization levels also affect the diet structure [16]. The present study, therefore also assesses the association between Body Mass Index (BMI)/Waist Circumference (WC) according to urbanization levels.

## 2. Methods

### 2.1. China Health and Nutrition Survey (CHNS)

CHNS is an ongoing open cohort longitudinal survey of nine waves (1989–2011). The survey uses a multistage random-cluster sampling process to select samples from nine provinces across China [17]. Since the 2000 survey, nine provinces across four regions were included: Northeast (Heilongjiang, Liaoning), East Coast (Shandong, Jiangsu), Central (Hennan, Hubei, Hunan) and West (Gunagxi, Guizhou), which covers all levels of socioeconomic development in China [18]. The selected provinces vary according to geography, economic development and health indicators. Within each province, counties were stratified by different income levels (low, middle, and high), and a weighted sampling scheme was used to randomly select four counties. The provincial capital and one other lower income city were selected when feasible, except that other large cities other than the provincial capitals had to be selected in two provinces. Within each county, one county capital town and three villages within the counties and urban/suburban neighborhoods within the cities were subsequently randomly selected. Finally, twenty households were randomly selected from within each village, town or neighborhood. All individuals in each household were interviewed in the CHNS. Details are described elsewhere [14,17]. In 2009, 15,866 participants aged 18 or over completed the survey. Of these, 2949 participants were aged 60 or above, and 2745 (93%) completed the dietary survey were included in the present study.

Survey protocols, instruments, and the process for obtaining informed consent for CHNS were approved by the institutional review committees of the University of North Carolina at Chapel Hill and the National Institute of Nutrition and Food Safety, China Centre for Disease Control and Prevention. All participants have given their written informed consent [17]. The University of Newcastle, Australia has also approved use of data in this study in 17 December 2013 (Approval Number: H-2013-0360).

### 2.2. Dietary Assessment and Food Grouping

Dietary assessment is based on a combination of three consecutive 24 h recall at the individual level, and a food inventory taken at the household level over the same three day period. Combination of consecutive three-day 24 h recall and household food inventory can improve the accuracy of recall [19]. Household food consumption was determined by weighing all food consumed by the household over three consecutive randomly selected days. The three consecutive days during which detailed household food consumption data have been collected were randomly allocated from Monday to Sunday and are almost equally balanced across the seven days of the week for each sampling unit. Household food consumption was determined by examining changes in inventory from the beginning to the end of each day, in combination with a weighing and measuring technique. All foods remaining after the last meal before initiation of the survey was weighed and recorded. All purchases as well as home production foods were recorded. Wasted food was estimated when weighing was not possible. At the end of the survey, all remaining food was again weighed and recorded.

To collect individual dietary data, each household member was asked to report all food consumed over the previous 24 h for each of the three days, whether at home or away from home. Interviewers recorded the types and amounts of food consumed at each meal during the previous day. The amount of food in each dish was estimated from the household inventory and the proportion of each dish consumed was reported by each person. Household food inventory was used to collect information of household level food intake and to further estimate the individual salt and oil intake. Extreme dietary data is based on the judgment of the interviewers. For example, if a person reported an intake of 2 kg of rice a day, this was regarded as extreme. Detailed dietary data collection is described elsewhere [14,17]. As the present study does not include calculation of salt and oil intake, we used data of 24 h recall over three consecutive days at the individual level for the analysis. The three-day recall method used in this study has a high correlation with the household food inventory method for each food group (e.g., correlation coefficient was 0.84 for rice, 0.84 for wheat) [19].

The food groups included were based on a food system developed specifically for CHNS and Chinese Food Composition Table [20]. Initially, 33 food groups were included. As some food items were consumed by less than 5% of participants, food intakes were further collapsed into 27 food groups based on similarity of nutritional profiles. The 27 food groups are: rice; wheat flour and wheat noodles; wheat buns and bread; corn and coarse grains; deep-fried wheat; starchy roots and tubers; pork; red meat; organ meat; processed meats; poultry and game; fish and seafood; milk; eggs and egg products; fresh legumes; legume products; dried legumes; fresh vegetables, non-leafy; fresh vegetables, leafy; pickled, salted or canned vegetables; dried vegetables; cakes; fruits; nuts and seeds; beer; liquor and fast food.

Mean consumption of each food group per day was calculated from dietary data, as liang (Chinese ounce, 1 liang = 50 g). Mean consumption of alcoholic beverages, soft drink, and tea was calculated from questionnaire responses. Respondents were asked “do you drink any kind of alcoholic beverage (beer or liquor)?”, and were asked further questions on drinking frequency, types and quantity consumed in a week. Also, participants were asked “do you normally drink tea?” and “do you drink soft drinks or sugared fruit drinks?” Further questions on drinking frequency and number of cups consumed per day (a cup is approximately 240 mL) were asked. Energy intake was calculated by CHNS based on Chinese Food Composition Table [21].

### 2.3. Outcome Variable-Body Composition

Height and body weight were measured based on a standard protocol recommended by the World Health Organization, by trained health workers. Weight in lightweight clothing was measured to the nearest 0.01 kg on a calibrated beam scale, and height was measured to the nearest 0.1 cm without shoes using a portable stadiometer [14,17]. BMI was divided into four categorical levels based on the criteria recommended by the Working Group on Obesity in China which are underweight: BMI < 18.5 kg·m^−2^; normal: BMI: 18.5–23.9 kg·m^−2^; overweight: BMI: 24.0–27.9 kg·m^−2^; general obesity: BMI ≥ 28.0 kg·m^−2^ [22]. Central obesity was defined as WC ≥90 cm in men and ≥80 cm in women according to the International Diabetes Federation criteria [6].

### 2.4. Other Variables

Education level was allocated into four categories from the six education categories in the questionnaire: illiteracy; low: primary school; medium; junior middle school and high; high middle school or higher. Marital status was categorized as married or other. Work status was divided into two categories (Yes/No). Smokers were identified as people who have at least one cigarette per day (Yes/No), based on the question “how many cigarettes do you smoke per day?” Drinking was allocated to two categories, with the question “last year, did you drink beer or any other alcoholic beverage?” Participants were asked about the time spent for different types of physical activities per week. We calculated Metabolic Equivalent of Task (MET) based on the Compendium of Physical Activities and CHNS [23,24]. Four types of physical activities were included to calculate the MET including domestic activity (e.g., buying food, cooking), occupational activity (e.g., light, moderate, and heavy), transportation activity (e.g., walking from work) and leisure activity (e.g., martial arts). Urbanization is defined by a multidimensional twelve-component urbanization index to capture population density and physical, social, cultural, and economic environments [14,25].

### 2.5. Statistical Analysis

We only analyzed cross-sectional data collected in 2009. Exploratory factor analysis was used to identify dietary patterns using principal component analysis method in STATA/SE 13.1 (STATA, StataCorp, College Station, TX, USA). The intake (liang or cups) of 27 food groups were included in the factor analysis. Dietary patterns were identified based on the eigenvalue (>1), scree plot, factor interpretability, and the variance explained (>5%). Factors were rotated with varimax to improve the interpretability of factors and minimize the correlation between factors.

Participants were assigned a pattern-specific factor score, which was calculated as the sum of the products of the factor loading coefficients and standardized daily intake of each food associated with that pattern. Factor loadings were included in the calculation of pattern scores.

Factor scores were divided into four quartiles based on their distribution in each stratum. As regions have different levels of socioeconomic development and are therefore a potential factor impacting on the nutrition status [18,26] we included urbanization level in the analysis. Mean and standard deviation (SD) across four quartiles were used to present the average consumptions in each quartile of each food item for each dietary pattern. ANOVAs were used to identify significant differences between the two dietary patterns across four quartiles of food intake. Multinomial regression models were used to examine the associations between BMI status and dietary patterns, stratified by gender. Poisson regression models, instead of logistic regression models [27,28], were used to examine the associations between central obesity (No/Yes) and dietary patterns, stratified by gender. Forest plots [29] were used to show the association between BMI/WC and each dietary pattern, stratified by gender and urbanization levels.

## 3. Results

Sample characteristics are shown in Table 1. Of 2745 participants with dietary data, 57% (*n* = 1563) were aged 60–69 years and 43% (*n* = 1182) were aged 70 or above. The prevalence of overweight/obesity was 37.5% for men and 42.4% for women. Women (67.5%) had a higher prevalence of central obesity than men (35.7%). 7% of participants (*n* = 198) aged 60 or over have no dietary data. Compared with those with dietary data, people with no dietary data were slightly older (mean: 71.1 *vs.* 69.3), and more lived in a rural area (74.8% *vs.* 63.5%).

**Figure 1:**
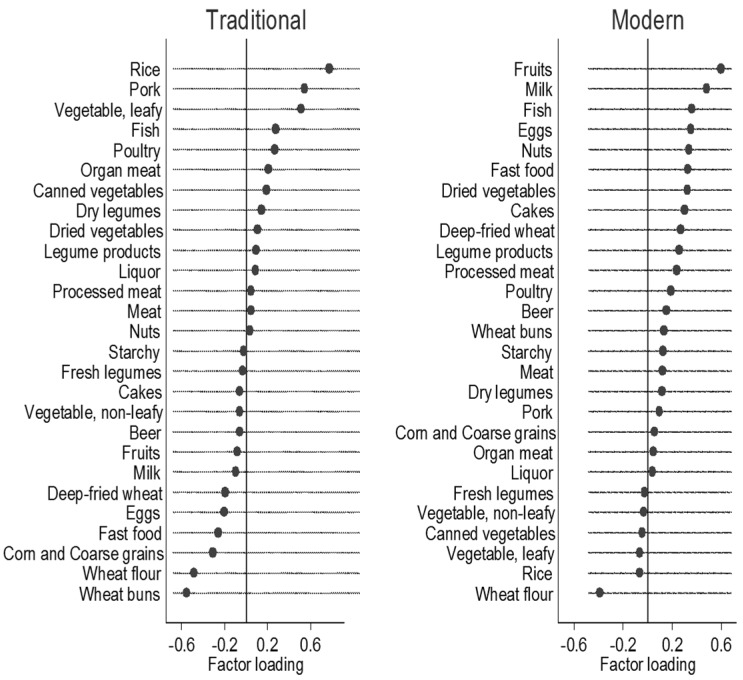
Factor loadings for two food patterns among older Chinese people (*n* = 2745) *. (* Factor loadings of >|0.20| represent the foods which most strongly related to the identified factor).

Two food patterns were obtained by factor analysis (Figure 1). Traditional dietary pattern (Eigenvalue = 2.25) was loaded heavily on rice, pork, and vegetables, and inversely on wheat flour and wheat buns. Modern dietary pattern (Eigenvalue = 1.75) is characterized by high intake of fruit, dairy, processed food, cakes and fast food, and inversely on rice and wheat flour. The two factors explained the 14.9% of variance in intake.

Table 2 shows food intakes across quartiles of traditional and modern dietary pattern, stratified by gender. For traditional dietary pattern, there were significant increases (*p* < 0.001) in rice, pork, fresh vegetable, fish, poultry and organ meat across quartiles for each gender. Significant decreases were found for corn, wheat flour, and wheat buns (*p* < 0.001). There was no statistically significant difference for energy intake of traditional dietary pattern for men (*p* = 0.14). Energy intakes were different across quartiles for women, but with no clear trend (*p* < 0.001). For the modern dietary pattern, there are significant increases (*p* < 0.001) in fruit, milk fast food, eggs, nuts, cakes, dried vegetables, fish, deep-fried wheat, processed meat, wheat buns, and legume products across quartiles for each gender. Significant decreases were found for rice and wheat flours (*p* < 0.001).

**Table 1:** Characteristics of study participants.

Factor	Men	Women	*p* Value	*N* (% of Participants)
	1300 (47.4%)	1445 (52.6%)		2745
**Age**				
Median (IQR)	67.0 (63.0, 74.0)	69.0 (63.0, 74.0)	0.06	2745 (100%)
**Physical activity (MET)**				
Median (IQR)	84.1 (62.4; 132.6)	101.8 (79.6; 133.3)	<0.001	2745 (100%)
**Marital status** *				
Married	1103 (85.6%)	926 (64.7%)	<0.001	2721 (99%)
Other marital status	186 (14.4%)	506 (35.3%)		
**Work status** *				
No	865 (66.9%)	1145 (79.6%)	<0.001	2731 (99%)
Yes	428 (33.1%)	293 (20.4)		
**Education level** *				
Illiteracy	176 (13.7%)	642 (44.8%)	<0.001	2721 (99%)
Low	573 (44.5%)	539 (37.6%)		
Medium	282 (21.9%)	140 (9.8%)		
High	256 (19.9%)	113 (7.9%)		
**Smoking status** *				
No	711 (54.9%)	1351 (93.6%)	<0.001	2739 (99%)
Yes	585 (45.1%)	92 (6.4%)		
**Urbanization**				
Low	455 (35.1%)	475 (32.9%)	0.39	2739 (99%)
Medium	424 (32.7%)	473 (32.8%)		
High	417 (32.2%)	495 (34.3%)		
**BMI** *				
Underweight	101 (8.2%)	121 (8.8%)	<0.001	2609 (95%)
Normal	669 (54.3%)	672 (48.8%)		
Overweight	380 (30.8%)	417 (30.3%)		
Obesity	83 (6.7%)	166 (12.1%)		
**Central obesity**				
No	834 (64.4%)	469 (32.5%)	<0.001	2739 (99%)
Yes	462 (35.7%)	974 (67.5%)		

* Significant differences have been found between physical activity, marital status, work status, education level, smoking status, BMI status and Central obesity (*p* < 0.001).

**Table 2:** Food intakes across qualities of traditional and modern dietary pattern for men and women.

Food items (Liang per day)	Q1		Q2		Q3		Q4		*p* for Trend
	Mean	SD	Mean	SD	Mean	SD	Mean	SD	
**Intake of traditional food pattern**									
**Men**									
Rice	1.36	1.64	3.89	2.07	5.22	2.07	8.01	3.65	<0.001
Pork	0.49	0.67	0.80	0.88	1.35	0.98	2.20	1.68	<0.001
Fresh vegetable, leafy	1.61	1.69	2.01	2.11	2.97	1.86	4.60	2.73	<0.001
Fish	0.18	0.55	0.54	0.97	0.78	1.15	1.12	1.58	<0.001
Poultry	0.11	0.37	0.13	0.46	0.21	0.54	0.48	0.94	<0.001
Organ meat	0.02	0.16	0.03	0.17	0.06	0.22	0.14	0.40	<0.001
Corn and coarse grains	1.03	1.76	0.41	1.07	0.23	0.72	0.11	0.42	<0.001
Wheat flour	3.66	3.27	1.37	1.37	1.21	1.13	0.57	0.91	<0.001
Wheat buns	2.94	2.87	0.99	1.68	0.37	0.81	0.17	0.47	<0.001
Oil (g)	60.4	219.8	45.0	65.3	44.8	45.5	52.5	140.5	0.35
Energy (kJ per day)	8542	8631	7940	3284	8679	2776	10616	6149	0.14
**Intake of traditional food pattern**									
**Women**									
Rice	3.89	2.07	3.52	1.84	4.79	1.77	7.19	3.31	<0.001
Pork	0.39	0.54	0.74	0.79	1.31	0.90	2.27	1.70	<0.001
Fresh vegetable, leafy	1.50	1.67	2.01	1.76	3.09	1.96	4.62	3.13	<0.001
Fish	0.13	0.41	0.36	0.76	0.69	1.06	1.11	1.74	<0.001
Poultry	0.05	0.20	0.11	0.38	0.23	0.54	0.51	0.92	<0.001
Organ meat	0.02	0.19	0.02	0.13	0.05	0.19	0.15	0.40	<0.001
Corn and coarse grains	0.91	1.70	0.34	0.85	0.15	0.48	0.12	0.52	<0.001
Wheat flour	2.87	2.51	1.25	1.24	0.88	0.97	0.47	0.76	<0.001
Wheat buns	2.25	2.27	0.65	1.19	0.27	0.66	0.19	0.55	<0.001
Oil (g)	52.4	178.7	37.3	39.9	39.2	28.5	53.9	157.0	0.82
Energy (kJ per day)	8140	6967	6797	2454	7442	2066	10616	6149	<0.001
**Intake of modern food pattern**									
**Men**									
Fruit	0.04	0.21	0.23	0.68	0.80	1.34	2.45	2.81	<0.001
Milk	0.0	0.0	0.0005	0.009	0.14	0.61	1.07	1.87	<0.001
Fast food	0.01	0.12	0.14	0.54	0.43	0.88	0.92	1.70	<0.001
Eggs	0.24	0.42	0.48	0.58	0.72	0.76	1.01	1.06	<0.001
Nuts	0.003	0.02	0.04	0.17	0.07	0.26	0.29	0.74	<0.001
Cakes	0.008	0.07	0.02	0.14	0.07	0.28	0.32	1.15	<0.001
Dried vegetable	0.009	0.06	0.01	0.07	0.06	0.20	0.16	0.41	<0.001
Fish	0.12	0.38	0.53	0.89	0.92	1.25	1.13	1.63	<0.001
Deep-fried wheat	0.003	0.04	0.03	0.15	0.15	0.39	0.55	1.22	<0.001
Processed meat	0.002	0.02	0.03	0.16	0.08	0.30	0.17	0.58	<0.001
Wheat buns	0.35	1.25	0.74	1.62	1.28	2.29	1.78	2.29	<0.001
Legume products	0.68	1.03	0.91	1.32	1.06	1.32	1.72	1.88	<0.001
Rice	5.91	4.29	5.29	3.12	4.62	3.43	3.63	2.87	<0.001
Wheat flour	2.85	3.29	1.50	1.67	1.35	1.57	0.95	1.34	<0.001
Oil (g)	54.3	136.4	42.0	30.4	55.5	161.0	24.6	170.0	0.85
Energy (kJ per day)	9440	6171	8460	2806	9447	6526	10231	6769	0.001
**Intake of modern food pattern**									
**Women**									
Fruit	0.07	0.33	0.33	0.80	0.74	1.31	3.28	3.36	<0.001
Milk	0.005	0.09	0.0012	0.016	0.13	0.55	1.21	1.94	<0.001
Fast food	0.01	0.10	0.12	0.46	0.47	0.85	0.97	1.80	<0.001
Eggs	0.19	0.36	0.49	0.55	0.67	0.63	0.94	0.88	<0.001
Nuts	0.006	0.05	0.03	0.13	0.05	0.18	0.20	0.56	<0.001
Cakes	0.001	0.02	0.02	0.12	0.06	0.23	0.25	0.73	<0.001
Dried vegetable	0.005	0.04	0.01	0.07	0.07	0.23	0.16	0.39	<0.001
Fish	0.12	0.39	0.38	0.71	0.66	1.10	1.06	1.68	<0.001
Deep-fried wheat	0.02	0.15	0.03	0.14	0.21	0.48	0.27	0.54	<0.001
Processed meat	0.005	0.04	0.03	0.15	0.05	0.20	0.13	0.39	<0.001
Wheat buns	0.19	0.75	0.69	1.41	1.41	2.08	1.25	1.63	<0.001
Legume products	0.55	0.80	0.73	1.12	1.16	1.42	1.42	1.81	<0.001
Rice	4.67	3.36	4.41	2.73	3.53	2.89	3.30	2.77	<0.001
Wheat flour	2.33	2.53	1.39	1.57	1.15	1.30	0.73	0.95	<0.001
Oil (g)	45.3	94.1	39.1	48.6	51.7	146.2	44.8	159.0	0.70
Energy (kJ per day)	7597	4375	7355	3096	8148	5849	8719	3504	<0.001

SD: standard deviation; liang: Chinese ounce, 1 liang = 50 g.

The prevalence of overweight and obesity was 29.4% and 8.4% in quartile 4 (Q4) and 34.9% and 11.3% in quartile 1 (Q1) of traditional dietary pattern. For the modern dietary pattern, compared with Q1, people in Q4 have a higher prevalence of being overweight and obese (Overweight: 36.7% in Q4, 23.3% in Q1; Obesity: 13.2% in Q4, 6.5% in Q1). Similar differences were found for central obesity. The prevalence of central obesity was 45.1% in Q4 and 59.0% in Q1 for traditional dietary pattern; while it was 59.1% in Q4, and 42.2% in Q1 for modern dietary pattern.

Multinomial and Poisson regression models are shown in Table 3. After adjusting for age, marital status, work status, education level, smoking, physical activity, modern diet pattern and energy (Table 3(a), model 2), men in the Q4 of traditional diet had relative risk ratios (RRRs) of 0.64 (95% CI: 0.45; 0.92) for overweight, and 0.66 (95% CI: 0.34; 1.27) for general obesity, compared with people in Q1. Across quartiles of traditional dietary pattern, RRRs for overweight/obesity decreased while RRRs for underweight increased. Similar results were found for women. Additionally, the Prevalence Ratios (PRs) for central obesity also significantly decreased across quartiles of traditional dietary pattern, especially for women (*p* for trend <0.001).

Modern dietary pattern was positively associated with general obesity for men and women (Table 3(b)). Compared with people with Q1 of modern dietary pattern, people with Q4 had higher RRRs for overweight/obesity; while people in Q4 had lower RRRs for underweight. Similarly, modern dietary pattern was positively, but traditional dietary pattern was inversely, associated with central obesity.

In order to confirm the associations, we also analyzed BMI and WC as continuous outcome variables by linear regression models. The results also show that traditional dietary pattern was inversely associated with BMI and WC, while modern dietary pattern was positively associated with BMI and WC. The association between dietary patterns and BMI was attenuated and became non-significant by adjusting for WC. However, the association between traditional dietary patterns and WC was independent of BMI (Appendix Table A1).

Furthermore, to minimize potential effects for other NCDs on BMI/WC, we performed an additional adjustment for NCDs (known diabetes, myocardial infarction and stroke), and the association between dietary pattern and BMI/WC did not change. Compared with Q1 of traditional dietary pattern, people in Q4 had lower BMI (β = −0.41, 95% CI: −0.56; −0.27) and lower WC (β = −1.70, 95% CI: −2.12; −1.29). Compared with Q1 of modern dietary pattern, people in Q4 had higher BMI (β = 0.35, 95% CI: 0.19; 0.51) and had higher WC (β = 1.43, 95% CI: 0.96; 1.90). After adjusting for three urbanization levels (model 3, Table 3), the *p* value changed dramatically, especially for the modern dietary pattern. This may imply that urbanization level is a potential confounding or matching variable. Data were thus stratified by urbanization for further analysis.

**Figure 2:**
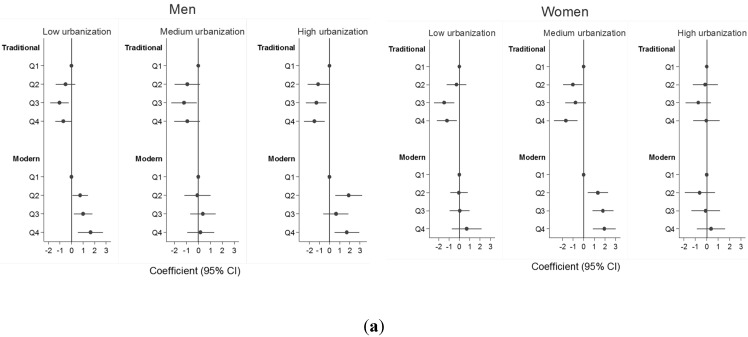

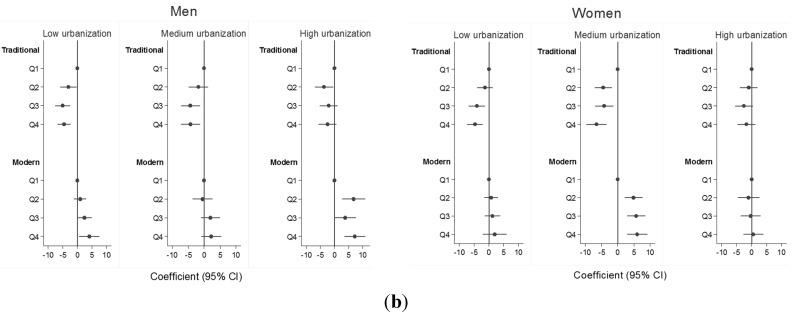
Dietary pattern and BMI/WC by gender, across three urbanization levels *. (* After adjusting for age, marital status, work status, education level, smoking, physical activity and energy). (**a**) Dietary pattern and BMI; (**b**) dietary pattern and WC.

Figure 2 shows the adjusted model of the association between BMI/WC (continuous variable) and quartiles of two dietary patterns, stratified by gender and three urbanization levels. The association between traditional dietary and BMI/WC were generally consistent across three urbanization levels for men and women. However, in women a positive association between modern dietary pattern and BMI/WC was only seen among those living in medium urbanization level.

**Table 3:** Relative Risk Ratios (RRRs)/Prevalence Ratios (PRs) and 95% confidence interval (CI) for traditional and modern dietary pattern, stratified by gender.

BMI	Q1	Q2	Q3	Q4	*p* for Trend
**(a) Intake of Traditional Dietary Pattern Quartiles**					
**RRRs**					
**Men**					
**Model 1**					
Normal	1	1	1	1	
Underweight	1	1.98 (0.97; 4.02)	1.89 (0.95; 3.76)	1.80 (0.91; 3.53)	0.36
Overweight	1	0.73 (0.51; 1.06)	0.61 (0.42; 0.87)	0.66 (0.47; 0.93)	0.002
Obesity	1	0.94 (0.50; 1.77)	0.61 (0.31; 1.18)	0.68 (0.36; 1.26)	0.26
**Model 2 ^a^**					
Normal	1	1	1	1	
Underweight	1	1.46 (0.69; 3.08)	1.64 (0.81; 3.32)	1.75 (0.87; 3.51)	0.20
Overweight	1	0.69 (0.47; 1.02)	0.58 (0.40; 0.84)	0.64 (0.45; 0.92)	0.001
Obesity	1	0.77 (0.39; 1.51)	0.48 (0.24; 0.98)	0.66 (0.34; 1.27)	0.20
**Model 3**					
Normal	1	1	1	1	
Underweight	1	1.55 (0.73; 3.31)	1.78 (0.87; 3.67)	1.91 (0.94; 3.86)	0.15
Overweight	1	0.65 (0.43; 0.96)	0.54 (0.36; 0.79)	0.61 (0.42; 0.87)	<0.001
Obesity	1	0.66 (0.33; 1.31)	0.40 (0.19; 0.83)	0.55 (0.28; 1.08)	0.09
**(a) Intake of Traditional Dietary Pattern Quartiles**					
**PRs**					
**Central obesity**					
**Model 1**					
No	1	1	1	1	
Yes	1	0.82 (0.66; 1.02)	0.69 (0.55; 0.86)	0.80 (0.66; 0.99)	0.01
**Model 2 ^a^**					
No	1	1	1	1	
Yes	1	0.82 (0.66; 1.02)	0.70 (0.55; 0.87)	0.82 (0.67; 1.01)	0.009
**Model 3**					
No	1	1	1	1	
Yes	1	0.78 (0.63; 0.98)	0.66 (0.52; 0.83)	0.80 (0.64; 0.97)	0.003
**Women**					
**Model 1**					
Normal	1	1	1	1	
Underweight	1	1.13 (0.62; 2.04)	1.90 (1.09; 3.32)	1.51 (0.83; 2.76)	0.18
Overweight	1	0.93 (0.67; 1.29)	0.83 (0.59; 1.17)	0.92 (0.64; 1.31)	0.28
Obesity	1	0.81 (0.52; 1.28)	0.72 (0.44; 1.15)	0.79 (0.48; 1.29)	0.05
**Model 2 ^a^**					
Normal	1	1	1	1	
Underweight	1	1.02 (0.55; 1.89)	1.75 (0.97; 3.14)	1.96 (1.03; 3.72)	0.05
Overweight	1	0.97 (0.69; 1.37)	0.86 (0.60; 1.23)	0.80 (0.55; 1.17)	0.06
Obesity	1	0.83 (0.52; 1.33)	0.72 (0.44; 1.19)	0.70 (0.42; 1.18)	0.02
**Model 3**					
Normal	1	1	1	1	
Underweight	1	1.12 (0.60; 2.10)	1.97 (1.08; 3.59)	2.14 (1.12; 4.11)	0.03
Overweight	1	0.91 (0.64; 1.30)	0.80 (0.55; 1.15)	0.76 (0.52; 1.11)	0.03
Obesity	1	0.77 (0.48; 1.25)	0.67 (0.40; 1.12)	0.68 (0.40; 1.14)	0.01
**PRs**					
**Central obesity**					
**Model 1**					
No	1	1	1	1	
Yes	1	0.87 (0.80; 0.96)	0.84 (0.76; 0.93)	0.82 (0.74; 0.92)	<0.001
**Model 2 ^a^**					
No	1	1	1	1	
Yes	1	0.88 (0.80; 0.96)	0.84 (0.76; 0.93)	0.81 (0.72; 0.90)	<0.001
**Model 3**					
No	1	1	1	1	
Yes	1	0.87 (0.79; 0.96)	0.84 (0.76; 0.93)	0.81 (0.72; 0.90)	<0.001
**(b) Intake of Modern Dietary Pattern Quartiles**					
**RRRs**					
**Men**					
**Model 1**					
Normal	1	1	1	1	
Underweight	1	1.16 (0.69; 1.95)	0.76 (0.42; 1.36)	0.41 (0.21; 0.82)	0.002
Overweight	1	1.44 (0.98; 2.12)	2.03 (1.40; 2.94)	2.17 (1.51; 3.13)	0.001
Obesity	1	2.83 (1.27; 6.33)	2.59 (1.14; 5.89)	4.28 (1.99; 9.21)	<0.001
**Model 2 ^b^**					
Normal	1	1	1	1	
Underweight	1	1.04 (0.60; 1.79)	0.88 (0.48; 1.63)	0.65 (0.31; 1.37)	0.07
Overweight	1	1.51 (1.01; 2.26)	1.86 (1.26; 2.76)	1.78 (1.20; 2.65)	0.05
Obesity	1	3.00 (1.31; 6.88)	2.02 (0.86; 4.75)	2.97 (1.31; 6.74)	0.02
**Model 3**					
Normal	1	1	1	1	
Underweight	1	1.03 (0.59; 1.78)	0.98 (0.52; 1.87)	0.78 (0.35; 1.73)	0.22
Overweight	1	1.43 (0.95; 2.15)	1.69 (1.12; 2.53)	1.58 (1.03; 2.41)	0.21
Obesity	1	2.59 (1.12; 6.00)	1.53 (0.64; 3.70)	2.07 (0.88; 4.90)	0.17
**PRs**					
**Central obesity**					
**Model 1**					
No	1	1	1	1	
Yes	1	1.13 (0.86; 1.48)	1.48 (1.15; 1.90)	1.89 (1.50; 2.39)	<0.001
**Model 2 ^b^**					
No	1	1	1	1	
Yes	1	1.15 (0.88; 1.50)	1.35 (1.04; 1.74)	1.60 (1.25; 2.04)	<0.001
**Model 3**					
No	1	1	1	1	
Yes	1	1.10 (0.84; 1.44)	1.24 (0.95; 1.60)	1.42 (1.10; 1.67)	<0.001
**Women**					
**Model 1**					
Normal	1	1	1	1	
Underweight	1	0.68 (0.42; 1.10)	0.81 (0.49; 1.33)	0.16 (0.06; 0.37)	<0.001
Overweight	1	1.04 (0.73; 1.48)	1.77 (1.25; 2.51)	1.70 (0.19; 2.42)	0.004
Obesity	1	1.02 (0.61; 1.70)	1.62 (0.98; 2.66)	2.00 (1.23; 3.25)	0.004
**Model 2 ^b^**					
Normal	1	1	1	1	
Underweight	1	0.64 (0.39; 1.06)	0.95 (0.56; 1.61)	0.21 (0.08; 0.52)	0.005
Overweight	1	1.01 (0.70; 1.45)	1.62 (1.12; 2.34)	1.43 (0.97; 2.12)	0.16
Obesity	1	0.96 (0.57; 1.62)	1.34 (0.80; 2.26)	1.47 (0.87; 2.51)	0.20
**Model 3**					
Normal	1	1	1	1	
Underweight	1	0.67 (0.41; 1.11)	1.06 (0.62; 1.83)	0.24 (0.09; 0.63)	0.03
Overweight	1	0.98 (0.68; 1.41)	1.50 (1.02; 2.18)	1.29 (0.86; 1.95)	0.43
Obesity	1	0.90 (0.53; 1.53)	1.21 (0.71; 2.08)	1.32 (0.76; 2.30)	0.37
**PRs**					
**Central obesity**					
**Model 1**					
No	1	1	1	1	
Yes	1	1.15 (1.02; 1.29)	1.18 (1.05; 1.32)	1.28 (1.14; 1.43)	0.001
**Model 2 ^b^**					
No	1	1	1	1	
Yes	1	1.14 (1.01; 1.28)	1.12 (1.00; 1.25)	1.19 (1.06; 1.33)	0.09
**Model 3**					
No	1	1	1	1	
Yes	1	1.13 (1.01; 1.27)	1.11 (0.98; 1.25)	1.18 (1.04; 1.33)	0.14

Model 1 crude model; Model 2 ^a^ adjusted for age, marital status, work status, education level, smoking, physical activity, modern diet pattern and energy; Model 2 ^b^ adjusted for age, marital status, work status, education level, smoking, physical activity, traditional diet pattern, and energy; Model 3 adjusted for urbanization levels and model 2.

## 4. Discussion

In this cross-sectional study, we found two distinct dietary patterns for older Chinese people. A traditional dietary pattern was inversely associated with the risk of overweight/obesity and central obesity, whilst positively associated with underweight. By contrast, a modern dietary pattern is significantly related to an increased likelihood of being obese and central obese, and inversely associated with underweight.

Although other dietary patterns have not been consistently identified in previous Chinese studies, the main components (rice, pork, and vegetable) of traditional dietary pattern were similar to those identified in this analysis [12,30]. The key components of traditional dietary pattern include high consumption of rice, pork, vegetables and fish, low consumption of meat, milk and ethanol, similar to the Mediterranean diet (protective against weight gain) [31,32]. We also found that traditional dietary patterns have protective roles for obesity/central obesity. The key components of modern dietary pattern include fruit, milk, processed and fast food. The benefits of intake of fruit are well documented and Chinese Nutrition Society recommends 200–400 g per day [33]. Although fruit is loaded heavily in the modern dietary pattern, the consumption amount is still below the recommended amount, and they are not likely to play a beneficial role in a healthy diet.

Rice is heavily positively loaded in the traditional dietary pattern, and loaded inversely in the modern dietary pattern. A previous systematic review among Asia populations indicated that rice with a high glycemic index was significantly associated with increased risk of developing type 2 diabetes [34]. However, there is still dispute about the association between rice intake and obesity within the Asian population [35,36]. In this study, a traditional dietary pattern that includes high intake of rice can be inversely associated with obesity. Our findings are consistent with studies done in Jiangsu province China, which found that a rice-rich traditional dietary pattern was inversely associated with weight gain [30,37]. As the prevalence of obesity and central obesity in the older Chinese population are high (Table 1), and these are very important biological risk factors for NCDs, our results imply the possibility of a further increase of obesity-related NCDs, such as diabetes, cardiovascular diseases and NAFLD [4,12]. Our study suggests that traditional dietary patterns may have inverse associations for obesity-related NCDs.

A positive association between modern dietary pattern and general/central obesity is consistent with current knowledge. As is shown in Table 2, fast food and processed meat intake increased substantially across quartiles for modern dietary pattern (*p* for trend <0.001). A modern dietary pattern is positively associated with fat and energy intake. The link between fat intake and obesity is well established [38]. However, fat and energy intake does not seem to explain the inverse association between traditional dietary pattern and obesity in the present study.

The discrepancy in the associations between modern dietary pattern and BMI/WC among women living in different urbanization levels may be because the composition of food in modern dietary pattern is different. One of our studies, which examined the association between dietary pattern and hypertension, found the different diet composition for modern dietary pattern at different urbanization levels. For example, the top loading foods are fruit and dairy across three urbanization levels, while the top loading foods are starchy and legumes in the low urbanization level [39]. Another possible reason may be that women living in high urbanization areas may be more concerned about body image and conscious about their health, and also undertake more physical activity than women from other areas.

Underweight is another public health concern among older Chinese people. We found that despite the rapid social and economic development in China, 8.2% of men, and 8.8% of women were still underweight in 2009. Older people are at risk of nutritional deficiency, which increases with age and the decline in a number of physiological functions. Diseases, medication, hospitalization, and other social determinants can also contribute to nutritional inadequacy [40]. It is crucial that dieticians and health professionals encourage older people to consume a healthy and balanced diet [15].

In addition, our results show that the traditional dietary pattern is inversely associated with BMI/WC. We also observed that there may be different food composition in the modern dietary pattern across different urbanization levels. Considering these results, the Chinese dietary guideline should be improved by encouraging healthy diets and the use of available healthy foods at the regional and local levels. Moreover, although the current Chinese guidelines provide general dietary advice to older people, age-specific dietary guidelines for the older Chinese population are needed in the prevention of obesity and NCDs [15,41].

Some limitations need to be addressed in the study. While urbanization level differences are important, the stratified analysis reported here was limited due to study power at the urbanization level. Further studies with larger sample sizes are needed. The association between dietary patterns and obesity may also be confounded by urbanization levels differences in other factors, which were not measured in this study. Although people who have been diagnosed with cancer may have low BMI as well as poor dietary intake, information on cancer was not available in CHNS. Moreover, the cross-sectional design in this study cannot draw conclusions about the etiological link between the two dietary patterns and obesity. However, these limitations do not affect the significance of the study. The present study breaks new ground by exploring dietary patterns and obesity among older populations in China. Strong gender and socioeconomic-specific evidence can inform policy makers and the development of programs for preventing obesity and NCDs in older Chinese people.

## 5. Conclusions

Prevention of obesity is vital in China, as obesity is a key risk factor for NCDs [7]. This study highlights the importance of a healthy diet for healthy ageing in China. Public awareness, clinical interventions, and nutrition policy are also need to recognize and plan for gender and geographical differences to maximize the success of health promotion approaches. Government should consider regulations and policies that encourage healthy diets and the availability of healthy foods, particularly at the regional and local levels. In addition, healthy dietary guidelines for older people should be developed, as this population group have specific dietary needs and are at higher risk of malnutrition [40].

## Appendix

**Table A1:** Associations between dietary patterns and BMI/WC.

Coefficients (95% CI)					
	Q1	Q2	Q3	Q4	*p* for trend
**(a) Intake of traditional dietary pattern quartiles**					
**BMI** *					
**Men**					
Mode1 1 ^a^	1	−0.79 (−1.35; −0.24)	−1.14 (−1.68; −0.61)	−0.92 (−1.43; −0.41)	<0.001
Model 1 ^a^ + WC		−0.01 (−0.38; 0.36)	−0.06 (−0.42; 0.30)	0.11 (−0.23; 0.45)	0.31
**Women**					
Mode1 1 ^a^	1	−0.66 (−1.22; −0.10)	−1.09 (−1.67; −0.51)	−1.06 (−1.66; −0.46)	<0.001
Model 1 ^a^ + WC		0.002 (−0.38; 0.39)	−0.09 (−0.49; 0.31)	0.11 (−0.31; 0.52)	0.96
**WC** *					
**Men**					
Model 1 ^a^	1	−3.13 (−4.81; −1.45)	−4.11 (−5.74; −2.48)	−3.81 (−5.35; −2.26)	<0.001
Model 1 ^a^ + BMI		−1.30 (−2.41; −0.18)	−1.74 (−2.82; −0.65)	−1.94 (−2.96; −0.91)	<0.001
**Women**					
Model 1 ^a^	1	−2.69 (−4.24; −1.14)	−3.76 (−5.36; −2.15)	−4.39 (−6.05; −2.72)	<0.001
Model 1 ^a^ + BMI		−1.17 (−2.23; −0.11)	−1.49 (−2.59; −0.38)	−2.19 (−3.33; −1.04)	<0.001
**(b) Intake of modern dietary pattern quartiles**					
**BMI** *					
**Men**					
Model 1 ^b^	1	0.81 (0.28; 1.34)	0.81 (0.26; 1.36)	1.26 (0.68; 1.85)	<0.001
Model 1 ^b^ + WC		0.38 (0.03; 0.73)	0.07 (−0.30; 0.43)	0.15 (−0.24; 0.54)	0.77
**Women**					
Model 1 ^b^	1	0.29 (−0.27; 0.85)	0.64 (0.05; 1.23)	1.04 (0.39; 1.69)	0.02
Model 1 ^b^ + WC		−0.04 (−0.42; 0.34)	0.11 (−0.30; 0.52)	0.27 (−0.18; 0.71)	0.68
**WC** *					
**Men**					
Model 1 ^b^	1	1.74 (0.15; 3.34)	2.72 (1.05; 4.40)	4.27 (2.51; 6.04)	<0.001
Model 1 ^b^ + BMI		−0.09 (−1.15; 0.97)	1.09 (−0.01; 2.20)	1.61 (0.44; 2.78)	<0.001
**Women**					
Model 1 ^b^	1	1.65 (0.10; 3.21)	2.33 (0.70; 3.97)	3.22 (1.42; 5.03)	0.002
Model 1 ^b^ + BMI		0.81 (−0.26; 1.87)	0.85 (−0.27; 1.97)	0.95 (−0.29; 2.19)	0.07

Model 1 ^a^ after adjusted for age, marital status, work status, education level, smoking, physical activity, modern diet pattern, energy and urbanization levels; model 1 ^b^ after adjusted for age, marital status, work status, education level, smoking, physical activity, traditional diet pattern, energy and urbanization levels; * BMI: Body Mass Index, * WC: Waist Circumference.
